# Cyclic-RGDyC functionalized liposomes for dual-targeting of tumor vasculature and cancer cells in glioblastoma: An *in vitro* boron neutron capture therapy study

**DOI:** 10.18632/oncotarget.16625

**Published:** 2017-03-28

**Authors:** Weirong Kang, Darren Svirskis, Vijayalekshmi Sarojini, Ailsa L. McGregor, Joseph Bevitt, Zimei Wu

**Affiliations:** ^1^ School of Pharmacy, University of Auckland, Auckland 1142, New Zealand; ^2^ School of Chemical Sciences, University of Auckland, Auckland 1142, New Zealand; ^3^ Australian Centre for Neutron Scattering, Australian Nuclear Science and Technology Organization, Sydney 2232, Australia

**Keywords:** boron neutron capture therapy (BNCT), dual-targeting drug delivery, glioblastomas, Integrin αv and β3, cyclic RGD peptides

## Abstract

The efficacy of boron neutron capture therapy depends on the selective delivery of ^10^B to the target. Integrins αvβ3 are transmembrane *receptors* over-expressed in both glioblastoma cells and its neovasculature. In this study, a novel approach to dual-target glioblastoma vasculature and tumor cells was investigated. Liposomes (124 nm) were conjugated with a αvβ3 ligand, cyclic arginine-glycine-aspartic acid-tyrosine-cysteine peptide (c(RGDyC)-LP) (1% molar ratio) through thiol-maleimide coupling. Expression of αvβ3 in glioblastoma cells (U87) and human umbilical vein endothelial cells (HUVEC), representing tumor angiogenesis, was determined using Western Blotting with other cells as references. The results showed that both U87 and HUVEC had stronger expression of αvβ3 than other cell types, and the degree of cellular uptake of c(RGDyC)-LP correlated with the αvβ3-expression levels of the cells. In contrast, control liposomes without c(RGDyC) showed little cellular uptake, regardless of cell type. In an *in vitro* boron neutron capture therapy study, the c(RGDyC)-LP containing sodium borocaptate generated more rapid and significant lethal effects to both U87 and HUVEC than the control liposomes and drug solution. Interestingly, neutron irradiated U87 and HUVEC showed different types of subsequent cell death. In conclusion, this study has demonstrated the potential of a new dual-targeting strategy using c(RGDyC)-LP to improve boron neutron capture therapy for glioblastoma.

## INTRODUCTION

Glioblastoma, also known as glioblastoma multiforme, is the most aggressive brain cancer with a short median survival of 15 months [1, 2]. Despite improvements in conventional therapies, including surgery techniques, chemotherapy developments and radiotherapy technologies, glioblastoma remains an incurable disease due to the poor specificity of current therapies and the high filtration and resistance features of the tumor tissue [3]. The ideal strategy for treatment is to selectively destroy glioblastoma while sparing the neighboring healthy tissues. Boron neutron capture therapy (BNCT) has emerged as a promising approach to selectively target glioblastoma [4].

BNCT is a binary treatment modality, combining systemic administration of a boron compound with local application of thermal neutrons [4]. Under low energy thermal neutron irradiation, boron-10 (^10^B), a non-toxic and non-radioactive constituent of the natural elemental boron, captures neutrons and the resultant fission reaction releases lethal and local radiant energy in the form of alpha (α) particles and lithium-7 (^7^Li) nuclei. The resulting α particles have an average high linear energy transfer value of 307 keV/μm [5] which can induce complex DNA double-strand breaks and consequent cell death [6]. These particles dissipate their kinetic energy over a distance of 5–9 μm, slightly less than the diameter of a single cell, therefore localizing the radiation damage to within, or close to, cells that bind or internalize ^10^B at sufficient concentrations [4, 7]. The minimum boron level, counted as ^10^B, required to generate lethal damage is estimated to be about 20 μg per gram of tissue or 10^9^ atoms per cell [8]. Therefore, the effectiveness and specificity of BNCT is largely dependent on the selective delivery of ^10^B in the tumor. By targeting the delivery of ^10^B to tumor cells, the neutron beams selectively destroy the boron-bearing tumor tissues leaving the surrounding normal tissues undamaged.

To date, two boron-containing agents, sodium borocaptate (BSH), and boronophenylalanine (BPA) have been clinically approved for the treatment of glioblastoma in the United States, Japan and Europe [4, 7, 9]. BSH (Na_2_B_12_H_11_SH) is a hydrophilic boron compound containing twelve ^10^B atoms per molecule, has low toxicity and high stability in biological medium [10]. However, the lack of tumor selectivity limited their clinical therapeutic outcomes. In the case of human glioblastoma, a disrupted and hyper-permeable blood–brain barrier (BBB) has been observed [11], the inter-endothelial fenestrations in tumors are reported to be up to 300 nm [12]. In this context, targeted boron delivery using nanotechnology has emerged as an attractive strategy to pass BBB and achieve glioblastoma-selective intracellular accumulation of ^10^B [13]. Through the attachment or encapsulation of boron compounds with nano-sized carriers (100-200 nm), such as liposomes [14, 15], nanotubes [16] and dendrimers [17], specific accumulation can be achieved by exploiting the leaky vasculature in the tumor and the resulting enhanced permeability and retention (EPR) effects [18]. In addition, the surface of these nanocarriers can be modified with specific ligands, such as transferrin [19, 20] or folate [21], to selectively bind receptors over-expressed in glioblastomas. These approaches have reportedly enhanced intracellular boron delivery and thus anti-tumor effects in preclinical studies.

Among these nanocarriers, liposomes are an attractive carrier for tumor targeting due to their amphiphilic properties, biocompatibility and ease for surface modification. These systems have no innate ability to recognize the vascular endothelial cells in malignant tumors and have limited transvascular transport [22]. Therefore, an approach is sought to increase the tumor selectivity by targeting the tumor neo-vasculature followed by enhanced tumor penetration.

It is well documented that integrins, such as αvβ3, are over-expressed in endothelial cells undergoing rapid angiogenesis as well as in some tumor cells but barely detectable in normal brain cells [23]. Overexpression of integrins αvβ3 have been observed in both tumor cells [24] and the microvasculature of glioblastoma [25]. Notably, glioblastomas are rich in microvasculature and characterized by rapid angiogenesis [26, 27]. Furthermore, the expression level of integrins in tumor vasculature correlates with the grade of malignancy of neuroblastoma [28, 29], making their vasculature a clinically important target. Peptides containing an arginine-glycine-aspartic acid (RGD) sequence are found to specifically bind with integrin αvβ3 [30]. Furthermore, compared to linear RGD peptides, cyclic RGD peptides, such as cyclic arginine-glycine-aspartic acid-tyrosine-cysteine (c(RGDyC)), are more stable and have higher affinity to integrin αvβ3 [31]. Both linear and cyclic RGD have been used as ligands for drug delivery to target tumors such as hepatoma and melanoma [32, 33]. Taken together, it was envisaged that cyclic RGD peptide-modified nano-sized boron delivery systems would provide a dual targeting approach by exploiting the overexpression of αvβ3 of both tumor vasculature and tumor cells of glioblastoma lead to more effective BNCT.

This study aimed to address the limited glioblastoma-specific tissue accumulation as well as the poor cellular penetration of the boron agent BSH, with a liposomal delivery system modified with c(RGDyC). The dual-targeting effect of c(RGDyC) modified liposomes to tumor vasculature and glioblastoma cells was evaluated on a representative tumor angiogenesis model, human umbilical vein endothelial cell line (HUVEC) [34]; and a glioblastoma cell model, human glioblastoma cell line (U87). Western Blotting was used to confirm the integrin αvβ3 expression on these cell lines and to compare with the human pancreatic carcinoma cell line MIA PaCa-2, the human breast cancer cell line MCF-7 and the mouse macrophage cell line RAW 264.7 to identify integrin-negative controls. Finally the *in vitro* BNCT efficacy of c(RGDyC) modified liposomes containing BSH was assessed on these cell lines by thermal neutron irradiation in comparison with liposomes without peptide modification and a BSH solution.

## RESULTS

### Formation of c(RGDyC) modified liposomes

A c(RGDyC) (1%, molar ratio) modified liposomal system (c(RGDyC)-LP) for the dual-targeting of tumor vasculature and glioblastoma cells was developed. The c(RGDyC) peptides were conjugated to the liposomal surface through a thiol-maleimide coupling reaction and a high attachment efficiency (>98%) was achieved following 24 h incubation at 22°C. A decrease in reaction temperature to 4°C resulted in no detectable attachment while an increase in temperature to 37°C resulted in 51.9% attachment efficiency. The successful conjugation at 22°C was confirmed by the observation that the zeta potential of liposomes dropped by 10 mV (p < 0.01) (Table 1).

**Table 1:** Particle concentration and stability of BSH loaded liposomes

Formulation	Liposome number per ml (× 10^13^)	^10^B atom number per liposome (× 10^8^)	Time (week)	Particle size (nm)	PDI	Zeta potential (mV)	BSH remain entrapped (%)
LP			0	123.9 ± 1.8	0.10 ± 0.02	−36.2 ± 0.6	100.0 ± 0.8
	5.57 ± 1.14	2.69 ± 0.49	2	122.0 ± 1.5	0.10 ± 0.02	−36.9 ± 2.5	100.7 ± 1.3
			4	133.5 ± 12.7	0.10 ± 0.04	−34.2 ± 1.5	89.2 ± 5.7
c(RGDyC)-LP			0	124.5 ± 1.2	0.07 ± 0.02	−46.0 ± 1.0	100.0 ± 2.6
	6.50 ± 0.55	2.23 ± 0.18	2	124.7 ± 1.6	0.07 ± 0.01	−45.3 ± 1.0	100.2 ± 0.8
			4	125.4 ± 1.3	0.07 ± 0.02	−46.7 ± 1.3	98.9 ± 1.8

Liposomes were stored in pellet form, protected from light and maintained at 4°C (mean ± SD, n=3).

### Characterization of liposomes

**Figure 1:**
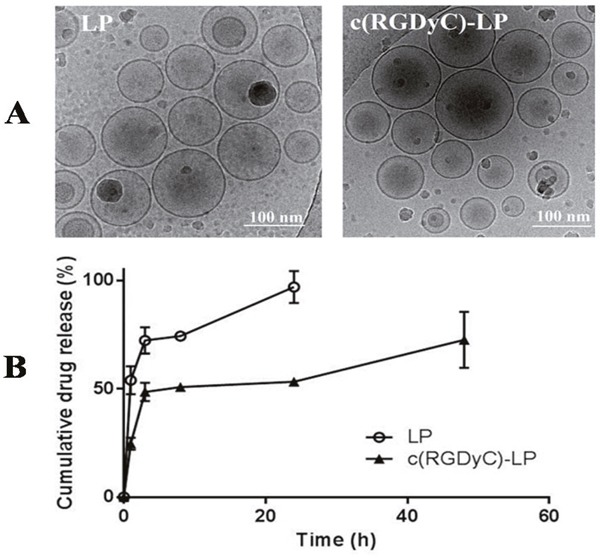
TEM images (A) and drug release profiles (B) from LP and c(RGDyC)-LP formulations Release studies were performed at 37°C in isotonic PBS (pH 7.4). Data are expressed as mean ± SD (n=3).

The unmodified (LP) and c(RGDyC) modified liposomes were around 124 nm in diameter. Except for zeta potential there was no significant change in particle physicochemical properties following conjugation with 1% (mol) c(RGDyC) (p > 0.05) (Table 1). Conjugation decreased the encapsulation efficiency (EE) of BSH within the liposomes from 5.5 ± 1.31% to 2.6 ± 0.04%, however the release of BSH was significantly slowed down (53.2 ± 1.3% versus 96.9 ± 7.4%) (Figure 1B). Cryo-TEM micrographs (Figure 1A) showed that c(RGDyC)-LP and LP were both unilamellar. The liposome concentration was found to be similar in the two formulations with 6.5 × 10^13^ /ml in c(RGDyC)-LP and 5.6 × 10^13^/ml in LP. Stability study demonstrated that the zeta potential, particle size and PDI of c(RGDyC)-LP remained unchanged and no drug leakage for at least 4 weeks, while drug leakage of LP was observed in week 4 (Table 1).

### Integrin αvβ3 expression analyzed by Western Blotting

**Figure 2:**
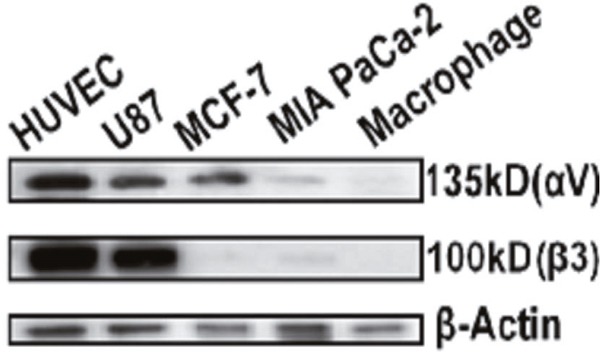
Western Blot analysis of cell expression of integrin αvβ3 HUVEC and U87 cells were confirmed to have strong expression of integrin αvβ3 while MCF-7 only express integrin αv, MIA PaCa-2 and macrophage cells had extremely weak expression of both αv and β3 subunits. β-actin was used as loading control.

Western Blotting analysis (Figure 2) indicated that HUVEC had the strongest expression of integrin αvβ3 followed by U87, while MIA PaCa-2 and macrophage cells did not display any detectable expression. Therefore, MIA PaCa-2 was used to represent normal cells with low expression of αvβ3 in the following studies. Interestingly, despite the wide report [35], breast cancer cell line MCF-7 only expressed the integrin subunit, αv.

### Determination of calcein working concentration

**Figure 3:**
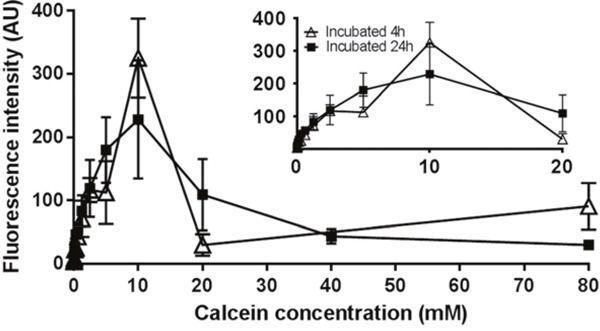
Fluorescence intensity after liposomes containing a series level of calcein were incubated with U87 cells for 4 h or 24 h Liposomes containing 10 mM of calcein generates the maximum fluorescence intensity. Results are expressed as mean ± SD (n=3).

A reversed U-shaped relationship between calcein concentration in liposomes and their fluorescence intensity in cells was observed after incubation of liposomes with U87 (Figure 3). Liposomes containing 10 mM calcein produced the maximum fluorescence and therefore were selected for the cellular uptake study, to ensure maximal fluorescence intensity and a positive correlation between the fluorescence signal and degree of uptake.

### Cellular uptake of liposomes

**Figure 4:**
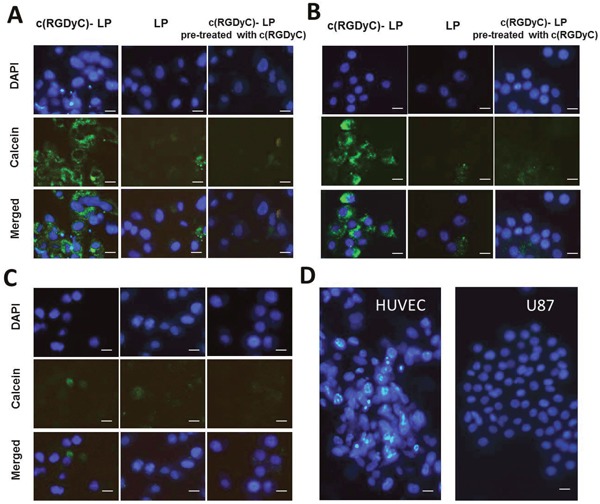
Representative fluorescence images showing selective cellular uptake of c(RGDyC)-LP corresponding to the expression of integrin αvβ3, which inhibited by pre-saturation with free c(RGDyC) In integrin αvβ3-positive HUVEC **(A)** and U87 cells **(B)**, c(RGDyC)-LP uptake is significantly higher than LP. No differential uptake was observed in integrin αvβ3-negative MIA PaCa-2 cells **(C)**. Cell aggregation was observed in c(RGDyC)-LP treated or c(RGDyC) pre-treated HUVEC and U87 cells **(D)**. DAPI was used to stain the nuclei (blue) and 10 mM calcein (green) was encapsulated in liposomes. Scale bar represents 10 μm. Cellular uptake of liposomes was recorded by fluorescence microscopy.

**Figure 5:**
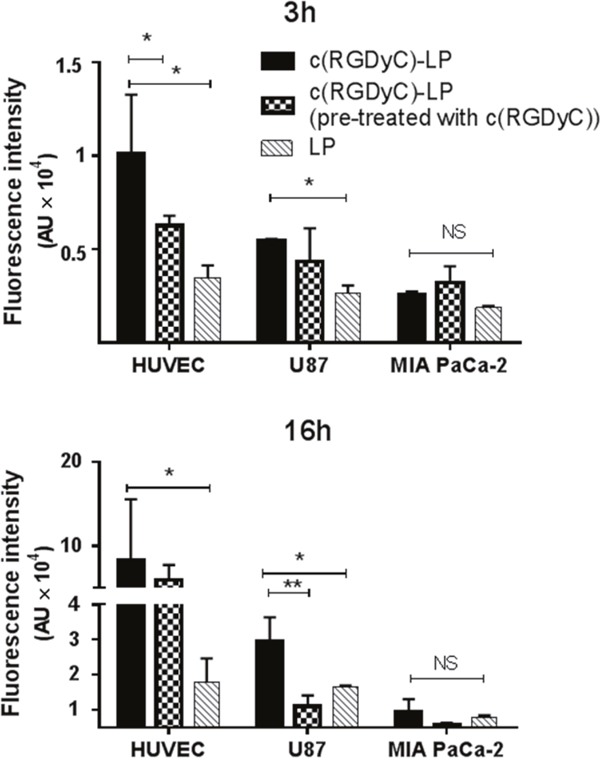
Cellular uptake of liposomes quantified by fluorescence intensity The difference in total uptake between liposomes with and without c(RGDyC) modification was statistically significant (p < 0.05) in HUVEC and U87 cells. Cells pre-treated with c(RGDyC) peptides were subsequently incubated with c(RGDyC)-LP. Cell aggregation in c(RGDyC)-LP treated HUVEC and U87 cells may have contributed to the relatively large error bars. Results are expressed as mean ± SD (*: p <0.05, **: p<0.01, and NS: p>0.05, n=3).

The degree of cellular uptake of c(RGDyC)-LP strongly correlated with the expression of integrin αvβ3 on the cell lines. In contrast to treatment with LP, treatment with c(RGDyC)-LP resulted in significantly higher levels of fluorescence in HUVEC and U87 cells (Figure 4A and 4B), but a similar level in MIA PaCa-2 (Figure 4C). The strong cellular uptake of c(RGDyC)-LP by the HUVEC and U87 cells was inhibited when pre-treated with free c(RGDyC) (Figures 4 and 5). The cellular uptake increased from 3 h to 16 h in all cases, the fluorescence intensity of c(RGDyC)-LP treated U87 cells was double that of LP treated cells at 3 h and maintained at the same ratio over 16 h. In HUVEC, the fluorescence intensity resulted from c(RGDyC)-LP treatment for 3 h was 2.5 times higher than the LP treatment, and the difference increased to > 4 times at 16 h (Figure 5). Notably, while the internalized fluorescence was distributed throughout the cytoplasm it was more prominent in the areas surrounding nuclei. Moreover, cell aggregation was observed in HUVEC and U87 cells (Figure 4D).

### *In vitro* BNCT

#### The effect of neutron irradiation on cell viability

**Figure 6:**
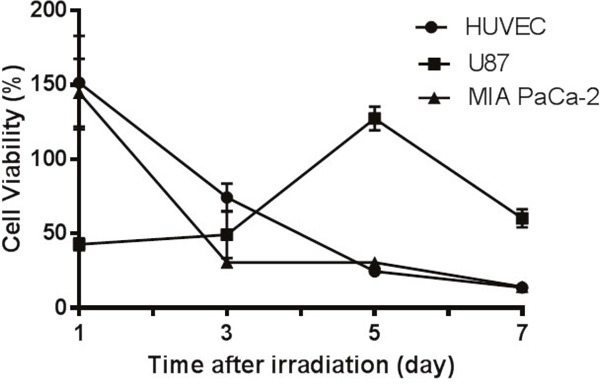
Cell responses to neutron irradiation in the absence of ^10^B HUVEC and MIA PaCa-2 cells underwent apoptosis after irradiation while glioblastoma cells U87 showed cell growth. The relative cell viability was obtained by comparing viability with non-irradiated cells maintained medium and monitored over 7 days after irradiation. Results are expressed as mean ± SD (n=3).

Figure 6 illustrates effect of neutron irradiation alone on HUVEC and U87 cells, expressed as the relative cell viability in comparison with non-irradiated cells (control). Irradiation appeared to stimulate HUVEC and MIA PaCa-2 cell metabolic activity initially resulted in a 150% relative cell viability at 24 h, however the cell viability declined continuously from day 1 with a 13% relative cell viability observed on the 7^th^ day. In contrast, neutron irradiation reduced the relative cell viability of U87 to 50% on day 1 and the cell viability maintained the same growth rate as the control cells up to day 3, however doubled at day 5 before the second drop at day 7.

#### The efficacy of BNCT on cell viability

**Figure 7:**
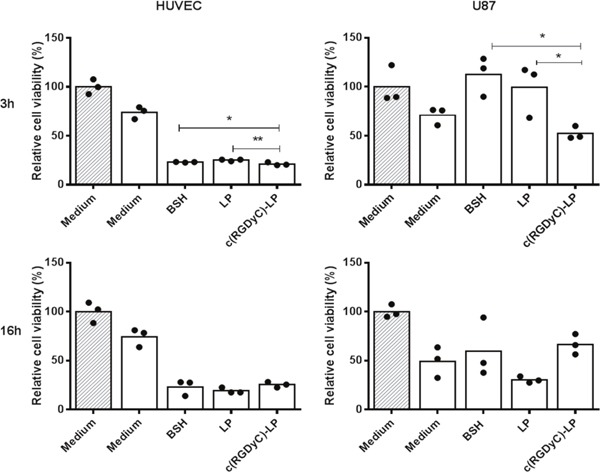
Efficacy of BNCT on cell viability of HUVEC and U87 cells Cells were pre-treated with different 10B containing formulations with the final concentration of 20 μg/ml 3 h or 16 h. The relative cell viability compared to non-irradiated cells maintained in culture medium was measured on the 4th day after irradiation by MTT assay. **: p<0.01, *: p<0.05. Filled columns are non-irradiated and blank columns are irradiated. The dots represent each of the individual data points.

Figure 7 shows the *in vitro* BNCT efficacy with the cells pre-treated with formulations for either 3 h or 16 h prior to 7 h irradiation. The cell viability measured on the 4^th^ day after irradiation was compared to non-irradiated control cells cultured in medium to demonstrate the BNCT efficacy. In both HUVEC and U87 cells with BNCT, the c(RGDyC)-LP pretreatment for 3 h led to the most significant reduction in cell viability compared with LP and BSH solutions. Extending the treatment with formulations to 16 h resulted in lower MTT cell viability close to 20% on HUVECs and 50% in U87 cells, regardless of the formulation (p>0.05). Moreover, U87 cell mutation was observed at day 3 post irradiation, some cells were giant shuttle-shaped and some were longer branched.

## DISCUSSION

In this study, we focused on a new approach by dual-targeting tumor vasculature and glioblastoma cells to enhance the efficiency of ^10^B delivery by exploiting the overexpression of integrin αvβ3 in both cell types. Hereby, a cyclic peptide c(RGDyC) modified liposomal delivery system has been developed and demonstrated to have dual-targeting potential.

With the optimised conditions, liposomes were covalently conjugated with c(RGDyC) with a high efficiency (> 98%) and within the optimal size range for exploiting the EPR effect (100-200 nm) [18]. The low EE of BSH in the nano-sized liposomes (approximately 5%) is typical for a water-soluble drugs like BSH, which predominately depends on the volume ratio of BSH solution inside and outside liposomes (V_in_/V_out_) during liposome formation [36, 37]. Therefore, EE can be enhanced by reducing V_out_ or increasing the mass of lipids (increasing V_in_), whereas the loading capacity could be further improved when a more concentrated BSH solution is used. The EE of BSH in c(RGDyC)-LP was reduced by 2% compared with LP, possible due to the drug leakage during the 24 h conjugation with the peptide. Conjugation with c(RGDyC) increased the magnitude of the zeta potential which is expected to increase the physical stability of liposomes through electrostatic repulsion. Besides, this negative charge would repel the ionized BSH species of the same charge, decreasing its release rate by diffusion across the liposomal membrane. This phenomenon has been reported to the other peptide-modified liposomes, such as asparagine-glycine-arginine (NGR) for doxorubicin and cyclic arginine-glycine-aspartic acid-tyrosine-lysine (c(RGDyk)) for cisplatin [38, 39].

Initially in this study, integrin αvβ3 expression was determined in several representative cell lines with both HUVEC and U87 showing strong expression of αvβ3 (Figure 2) which is consistent with the literature. Little or no expression was found in other cancer cell lines tested, MIA PaCa-2 cells and MCF-7 cells. The degree of cellular uptake of c(RGDyC)-LP strongly correlated with the cellular expression of integrin αvβ3 (Figure 4). The MIA PaCa-2 cancer cell line, with no integrin αvβ3 expression, showed no benefit to use c(RGDyC)-LP. The study suggests that evaluation of biomarker expression in the target tissues is necessary for developing personalized targeted therapies. In integrin-positive HUVEC and U87 cells, c(RGDyC) significantly enhanced the cellular uptake of c(RGDyC)-LP most likely through integrin αvβ3-mediated pathways. This is also supported by the finding that pretreatment with c(RGDyC) inhibited the cellular uptake of c(RGDyC)- LP. However, αvβ3-mediated endocytosis is not the only pathway for the internalization of c(RGDyC)-LP. Cao *et al*. reported that other endocytic pathways including macropinocytosis and clathrin-dependent endocytosis were also involved in the cellular uptake of RGD modified liposomes [40]. Interestingly, HUVEC and U87 cells which have strong integrin αvβ3 expression aggregated when treated with c(RGDyC)-LP, or following pretreatment with free c(RGDyC) (Figure 4). Besides, the aggregated cells tended to detach from the wells, resulting in large variant in fluorescent intensities (Figure 5). This could be explained as c(RGDyC) occupied the cell surface integrins, therefore blocked integrin-mediated cell adhesion and led to cell detachment and even apoptosis [29, 41].

To understand *in vitro* BNCT, the effect of neutron irradiation on cells without formulation pretreatment was first investigated. Following irradiation different cells appeared to have different response in the following 7 days (Figure 6). Neutron radiation can induce cell deaths by two pathways, apoptosis and necrosis [42]. Cell necrosis may be reflected by MTT assay directly, while apoptotic cells retain ability to reduce MTT salts in the early stage after irradiation [43]. The cell growth curves may indicate that HUVEC and MIA PaCa-2 cells underwent apoptosis with an initially high metabolic activity (relative viability ~150% at day 1 for both); but as apoptosis further programmed MTT absorbance dropped over time. On the other hand, U87 cells may have experienced necrosis in response to irradiation, and lost the viability (50%) rapidly as observed at day 1. However, as a type of brain tumor cells, U87 cells have higher capacity of DNA repair [44]. This may lead to rapid proliferation as observed on day 3 and day 5. The cells with unrepaired DNA break leads to subsequent cell death which occurs after a variable number of cell cycles [45], explaining the low MTT response on day 7. On the other hand, the cells with misrepaired DNA will lead to mutation [45]. Indeed, mutant U87 cells were also observed under microscope from day 3 to day 7.

Based on the above understanding of the cell lines, an MTT assay was carried out on day 4 in the *in vitro* BNCT study, as on this time both necrosis and apoptosis could be observed for all cells. The MTT assay which assesses cell metabolic activity was used to quantify BNCT effects in this study as it is prescribed as a quantitative cytotoxicity technique in the latest revision of ISO International Standards [46]. Furthermore, MTT assay has been employed to determine cell survival after irradiation [47, 48] with a reported similarity to clonogenic assays [49].

In the *in vitro* BNCT study, pretreatments of HUVEC with all formulations for only 3 h resulted in 70-80% cell death, with c(RGDyC)-LP being more significant than LP and BSH solution (Figure 7). The conventional liposomes can be internalized through clathrin-mediated endocytosis. Cyclic RGD peptides are reported not only able to bind to integrin αvβ3 as a ligand but also recruit clathrin and thus promote clathrin-mediated endocytosis [50, 51]. Hence, the superiority of the use of c(RGDyC)-LP over LP and BSH was observed with 3 h pretreatment. When incubation time extended to 16 h, all the formulations could have delivered sufficient amount of ^10^B to lead to complete cell death after irradiation. With U87, 3 h pretreatment saw a significant reduction in MTT viability measured at the day 4 with c(RGDyC)-LP than LP or BSH solution treatment. The high variations of the data at day 4 may be due to misrepaired or unrepaired DNA as the result of irradiation, and these cells would possibly undergo subsequent apoptosis, as shown in Figure 6.

Other researches pre-incubated cells with ^10^B formulations for up to 24 h prior to BNCT [47, 52]. This research suggests that even 3 h pretreatment with c(RGDyC)-LP was sufficient to generate lethal effects for both HUVEC and U87. Given the dynamic change of liposomes in the bloodstream, 3 h pretreatment in the *in vitro* study may be more clinically relevant.

In summary, the findings from this study has highlighted the potential of a new dual-targeting approach using c(RGDyC) modified liposomes for specific boron delivery to glioblastoma, addressing the major limitation of poor tumor accumulation of ^10^B in BNCT. Rather than targeting the tumor cells alone, the distinctive features of glioblastoma, rich microvascularture has also been exploited. To confirm our novel dual-targeting strategy, translational research with precise tracking of liposomal boron uptake by tumor using animal model is of great interest for future research.

## MATERIALS AND METHODS

### Materials

Sodium borocaptate (BSH) was purchased from Katchem Limited (Czech Republic) and c(RGDyC) from GL Biochem Limited (Shanghai, China). The phospholipids, 1,2-dipalmitoyl-sn-glycero-3-[phospho-rac-(1-glycerol)] (sodium salt) (DPPG), N-[(3-maleimide-1-oxopropyl)aminopropyl polyethyl eneglycolcarbamyl] distearoylphosphatidyl-ethanolamine (DSPE-PEG_2000_-MAL) and 1,2-distearoyl-sn-glycero-3-phosphoethanolamine-N-[methoxy(polyethylene glycol)-2000] (DSPE-mPEG_2000_) were purchased from Avanti Polar Lipids, USA. Cholesterol (99% pure), 4',5′-bis [N,N-bis(carboxymethyl)aminomethyl] fluorescein (calcein) and 2-(4-amidinophenyl)-6-indolecarbamidine dihydrochloride (DAPI) were obtained from Sigma-Aldrich (Auckland, New Zealand). All the chemicals used for HPLC were of analytical grade from EMD Millipore Corporation, USA. Water purified on a Milli-Q system (Millipore, USA) was used. The human brain glioblastoma cell line U87 (gift from the Auckland Cancer Society Research Centre) was cultured in minimum essential media (Life Technologies, CA, USA) supplemented with 10% fetal bovine serum (FBS, New Zealand origin, Life Technologies, Auckland, New Zealand) and 1% penicillin-streptomycin-glutamine (PSG, Life Technologies, CA, USA). The human umbilical vascular endothelium cell line HUVEC was purchased from Invitrogen (CA, USA) and cultured in medium 200 (Life Technologies, CA, USA) with the addition of 2% low serum growth supplement (Life Technologies, CA, USA), 20% of FBS and 1% of PSG. Human breast cancer cell line MCF-7 (ATCC, VA, USA), human pancreatic cancer cell line MIA PaCa-2 (gift from the Auckland Cancer Society Research Centre) and a mouse macrophage cell line RAW 264.7 (ATCC, VA, USA) were cultured in dulbecco's modified eagle's medium (Life Technologies, CA, USA) supplemented with 10% FBS and 1% PSG. All cell lines were maintained in an incubator with 5% CO_2_/95% air at 37°C. Protease inhibitor cocktail tablets were purchased from Roche, Basel, Switzerland. All the other chemicals for Western Blotting were obtained from Bio-rad (Hercules, CA, USA).

### Formation of c(RGDyC) modified liposomes

Plain liposomes were first prepared, PEGylation was applied to liposomes to avoid being cleared from the blood stream before reaching the tumor cells [53]. Liposomes contain DSPE-PEG_2000_-MAL could then be conjugated with c(RGDyC) to form c(RGDyC) modified liposomes.

#### Preparation of plain liposomes

Plain liposomes were prepared by the thin-film-hydration method. DPPG, cholesterol, DSPE-mPEG_2000_ and DSPE-PEG_2000_-MAL (molar ratios 6.5:3:0.4:0.1) were dissolved in chloroform: methanol (3:1, v/v) in a round-bottom flask. The solvent was gently removed on a rotary evaporator under reduced pressure to form a thin lipid film on the flask wall which was then put under nitrogen to completely remove traces of organic solvent. The thin lipid film was hydrated at 45°C in 50 mM BSH, or calcein dissolved in 100 mM HEPES buffer (pH 7.4). Seven cycles of freeze-thaw were applied after liposome formation involving freezing in liquid nitrogen and then thawing in a 45°C water-bath [54]. The resulting liposomes were extruded through 200 nm membranes to obtain a uniform size.

Control liposomes without c(RGDyC) modification (LP) composed of DPPG, cholesterol, DSPE-mPEG_2000_ (molar ratios 6.5:3:0.5) were also prepare as described above.

#### Attachment of c(RGDyC)

The c(RGDyC) was subsequently grafted to the surface of liposomes through a thiol-maleimide coupling reaction of c(RGDyC) with DSPE-PEG_2000_-MAL. Briefly, c(RGDyC) (1%, molar ratio) was dissolved in 100 mM HEPES buffer (pH 7.4) and incubated with the liposomes for 24 h at 4, 22 or 37°C to determine the optimal condition for maximum attachment. Nitrogen was inserted during incubation to avoid oxidization. A c(RGDyC) standard solution at the same conditions was used as a reference (%) for analysis.

#### Measurement of c(RGDyC) attachment efficiency

Unconjugated free c(RGDyC) was separated from liposomes by ultra-centrifugation at 41,000 rpm for 1 h at 4°C and quantified by HPLC analysis. The attachment efficiency (%) was calculated by comparing the peaks of the c(RGDyC) in the supernatant with a c(RGDyC) standard solution. All the analysis were carried out using a Phenomenex C_18_ (5 μm, 250 mm × 4.60 mm) column maintained at 40°C based on a gradient HPLC method modified from previous report with minimal modification [55]. Briefly, the mobile phase at a constant flow rate of 1 mL/min consists of 0.1% trifluoroacetic acid in water (eluant A) and 0.1% trifluoroacetic acid in acetonitrile (eluant B). The gradient elution was set from 10% to 50% B in 50 min, and back to 10% B over 5 min. The detection wavelength was set at 280 nm. The retention times of free c(RGDyC) and its dimer were 10.3 and 14.9 min, respectively.

### Characterization of liposomes

The size, polydispersity index (PDI) and zeta potential of the liposomes were measured before and after c(RGDyC) attachment by dynamic light scattering using a Zetasizer Nano-ZS (Malvern Instruments, UK). All measurements performed in triplicate at 25°C.

To determine the EE of BSH, free drugs and liposomes were separated by ultra-centrifugation at 41,000 rpm for 1 h at 4°C. The liposomes were re-suspended after centrifugation, and the encapsulated BSH was extracted from liposomes using acetonitrile (1:4, v/v). The concentrations of BSH were analysed by a validated ion-pairing HPLC method using a Phenomenex C_18_ column (5 μm, 250 mm × 4.60 mm) with UV detection at 230 nm. A ternary mobile phase system consisted of methanol, acetonitrile and 10 mM tetrabutylammonium hydrogensulfate buffer (pH 7) (30:30:40, v/v/v). The EE was calculated as the amount of BSH encapsulated in the liposomes versus the total quantity of BSH added for preparation. The particle concentration was determined using Nanosight nanoparticle tracking analysis (NanoSight NS300, Malvern, Worcestershire, UK) at camera level of 10. Data was analyzed on the NTA software 3.0 (ATA Scientific, Australia).

To investigate the drug release properties, pelletized liposomes were suspended in isotonic phosphate-buffered saline (PBS, 0.01 M, pH7.4) and maintained at 37°C with gentle shaking to simulate the *in vivo* conditions. Samples were taken at 1, 3, 8, 24 and 48 h. Released drug was immediately isolated by centrifugation at 41,000 rpm for 1 h at 4°C, and subjected to HPLC analysis. The amount of drug released at 4°C during the centrifugation was negligible. All the samples were placed in closed Eppendorf tubes sealed by Parafilm to avoid evaporation and kept in dark.

For stability tests, liposomes in pellet form were stored in the dark at 4°C. Particle size, PDI, zeta potential and drug leakage were monitored over 30 days.

The morphology liposomes were investigated using cryogenic transmission electron microscopy (cryo-TEM). The liposomes were diluted ten times with isotonic PBS (0.1 M, pH 7.4) and placed on a copper grid in the climate chamber and blotted, leaving a thin film stretched over the holes. The samples were frozen by submersion in liquid ethane and cooled to 90 K by liquid nitrogen. Samples were exposed to electrons and photographed at an accelerating voltage of 120 kV in a Tecnai 12 transmission electron microscope (FEI, Hillsboro, USA).

### Western blotting

Western Blotting was used to detect the expression of integrin αvβ3 on the various cell lines, HUVEC, U87, MIA PaCa-2, MCF-7 and RAW 264.7. Each cell line (10^6^ cells) was washed 3 times with ice-cold isotonic PBS (0.01M, pH 7.4) and protein extracts were prepared by lysing in NP40 cell lysis buffer (Life Technologies, USA) on ice. The lysates were then centrifuged at 13,400 rpm for 10 min; the supernatant was collected and mixed with loading dye. The mixture was heated at 95°C for 5 min before loading into a 10% Mini-PROTEAN TGX precast protein gel. Electrophoresis was performed in a vertical electrophoresis system with Tris-glycine running buffer (25 mM Tris, 190 mM glycine, 0.1 % SDS, pH 8.3). The proteins were allowed to separate for 30-40 min at 200 V and then transferred into a PVDF membrane using the Transblot turbo™ transfer system (Bio-Rad, Hercules, CA, USA). Membranes were blocked with 5% skim milk (Select, New Zealand) in 0.01 M PBS (pH 7.4) for 1 h at room temperature and then probed with primary antibodies, integrin rabbit anti-human αv antibody, rabbit anti-human integrin β_3_ antibody (both at 1:1000, Cell Signaling, MA, USA) or a goat anti-human β-actin antibody (1:500, Santa Cruz, TX, USA), overnight at 4°C. Thereafter membranes were incubated with an HRP-labeled goat anti-rabbit secondary antibody (1:5000/1: 10000, Cell Signaling, MA, USA) for 1 h at room temperature. Bolts were finally developed with electrochemiluminescence prime substrate (GE Healthcare, Buckinghamshire, UK) and imaged for chemiluminescence using a Fujifilm LAS-3000 imager.

### Cellular uptake study

#### Determination of calcein working concentration

Calcein, a hydrophilic fluorescent marker, was used to evaluate the interaction between liposomes and target cells [56, 57]. Its self-quenching properties at high concentrations resulting in a nonlinear signal-concentration [58] necessitated the determination of an optimised working concentration in liposomes. Briefly, liposomes were prepared as described above, with calcein solution being used to hydrate the lipid thin film. After seeding cells in a 96-well plate at a density of 3,000/100 μL/well for 24 h, U87 cells were incubated with 200 μL of liposomes containing different levels of calcein for 4 h or 24 h at 37°C. After washing the cells with isotonic ice-cold 0.01 M PBS (pH 7.4) three times, the fluorescence intensity of remaining intracellular calcein was measured in a microplate reader (λ _ex_/λ _em_: 495/515 nm, SpectraMax M2, Molecular Devices, CA, USA). The concentration which provided maximal fluorescence intensity was selected for the subsequent cellular uptake study.

#### Cellular uptake of liposomes

The cellular uptake of calcein-encapsulated liposomes with or without 1% c(RGDyC) on HUVEC and U87 cells in comparison with MIA PaCa-2 cells was observed by fluorescence microscopy and quantified by measuring the fluorescence intensity, as described above, following 3 h or 16 h incubation. In parallel, a competition assay was performed by pre-incubating cells with excess of c(RGDyC) (0.5 mM) for 15 min before cellular uptake was observed.

For microscopy imaging, the cells were fixed by adding 4% paraformaldehyde to each well. After rinsing with PBS (0.01 M, pH 7.4), 300 nM of DAPI (λ _ex_/λ _em_: 345/661 nm) staining solution was added to stain the nucleus acids. Cells were visualized using a standard fluorescence microscope (Leica DU IL LED, Danaher, Wetzlar, Germany).

### *In vitro* BNCT

The *in vitro* neutron irradiation experimental procedures were modified from reported studies [59, 60]: cells seeded in 96-well plates at a density of 3,000/100 μL/well were cultured for 24 h, and 200 μL of each of the formulations suspended in medium at a final equivalent concentration of 20 μg/mL ^10^B. Following 3 h or 16 h incubation, cells were washed three times and 450 μL fresh medium was added to fully fill each well. Irradiation was conducted by placing the sealed plates vertically on the Dingo thermal neutron radiography/tomography/imaging station sample stage and irradiating for 7 h at room temperature to impart a total neutron fluence of 1.2 × 10^12^ neutrons/cm^2^ in high-flux configuration following the published neutron irradiation studies [48, 61]. Meanwhile cells without irradiation were kept under the same conditions acted as controls. The Dingo instrument is located at the 20 MW OPAL research reactor, Australian Nuclear Science and Technology Organization (Sydney, Australia). The actual thermal neutron and photon dose at the sample position was measured from the radioactivation of pure gold foils placed at multiple spatial positions on the front side of each plate, and low neutron sensitivity thermoluniscent (LiF;Mg,Ti) (TLD-700) dosimeters placed on each side of the plate [62]. The Cadmium Ratio Method was used to obtain the absolute thermal flux values and doses delivered by BNCT to the cells [63]. The thermal neutron flux and photon dose rate imparted to the plates were 4.5 × 10^7^ n cm^−2^ s^−1^ and 0.277 mGy s^−1^, respectively. A 6% decrease in neutron flux was recorded across the well plate, corresponding to the radial distribution of the collimated neutron beam at the sample position (L/D = 500).

After irradiation, cell survival and growth were monitored over 7 days using a MTT cell viability assay [49]. The non-irradiated cells cultured in medium only were used as control to obtain relative cell viabilities for the treated cells. Cell growth curves over 7 days after irradiation were created to demonstrate their radiosensitivity to neutron irradiation alone. Histograms of relative cell viability in the 4^th^ day were generated to illustrate the BNCT efficacy of different formulations.

### Statistical analysis

Data were analysed by multiple t-tests using GraphPad Prism 6, version 6.01 (GraphPad Software, Inc.). P values less than 0.05 were considered as statistically significant, while those less than 0.01 were considered as highly significant.

## Abbreviations

BNCT: boron neutron capture therapy
RGD: arginine-glycine-aspartic acid
c(RGDyC): cyclic arginine-glycine-aspartic acid-tyrosine-cysteine peptide
BSH: sodium borocaptate
BPA: boronophenylalanine
BBB: blood–brain barrier
EPR: enhanced permeability and retention effects
LP: plain liposomes
c(RGDyC)-LP: c(RGDyC) modified liposomes
EE: encapsulation efficiency
PDI: polydispersity index
MTT: 3-(4,5-dimethylthiazol-2-yl)-2,5-diphenyltetrazolium bromide
DPPG: 1,2-dipalmitoyl-sn-glycero-3-[phospho-rac-(1-glycerol)] (sodium salt)
DSPE-PEG_2000_-MAL: N-[(3-maleimide-1-oxopropyl)aminopropyl polyethyl eneglycolcarbamyl] distearoylphosphatidyl-ethanolamine
DSPE-mPEG_2000_: 1,2-distearoyl-sn-glycero-3-phosphoethanolamine-N-[methoxy(polyethylene glycol)-2000]
Calcein: 4',5′-bis [N,N-bis(carboxymethyl)aminomethyl] fluorescein
DAPI: 2-(4-amidinophenyl)-6-indolecarbamidine dihydrochloride
FBS: fetal bovine serum
PSG: penicillin-streptomycin-glutamine
SD: standard deviation
